# MALDI-TOF MS profiling approach: how much can we get from it?

**DOI:** 10.3389/fpls.2015.00184

**Published:** 2015-03-23

**Authors:** Angela Mehta, Luciano P. Silva

**Affiliations:** Embrapa Recursos Genéticos e Biotecnologia, Parque Estação BiológicaBrasília, Brazil

**Keywords:** MALDI-TOF MS profiling, plants, microorganisms

Mass spectrometry has brought unprecedented possibilities in the field of proteomics. The advances obtained in the last 10 years have been outstanding and have enabled faster and more reliable data acquisition and comparison. One powerful method developed was the matrix-assisted laser desorption/ionization time-of-flight mass spectrometry profiling (MALDI-TOF MS profiling) approach, primarily used for the rapid and accurate identification of microorganism species. At least three commercial manufacturers developed systems (instrument, processing method, and databases) for this purpose with relatively similar results although not completely comparable or exchangeable. These methods were conceived for species level identification; however we explored these approaches with some few modifications for expression profiling purposes. We tested different samples, from diverse organisms (not only microorganisms) and found that MALDI-TOF MS profiling methods also have the ability to differentiate samples submitted to different biological conditions (e.g., biotic or abiotic stresses). In the case of MALDI-TOF MS profiling traditional approach, the extraction procedure is based on the enrichment of ribosomal proteins. By using different extraction protocols, samples can also be enriched with different types of proteins. Indeed, when proteins considered for profiling were further analyzed by MALDI TOF/TOF methods, other proteins could also be detected. Although MALDI-TOF MS profiling methods have been used in several tissues and samples, this approach has been rarely employed in plants. One of the applications in plant research is the identification of biomarkers associated to disease, which could, for example, help on quarantine procedures. Overall, MALDI-TOF MS profiling has a high potential to contribute for sample differentiation and biomarker identification and should be better explored in plant proteomics.

## Advances and applications of MALDI-TOF MS profiling

Matrix-assisted laser desorption/ionization time-of-flight mass spectrometry profiling (MALDI-TOF MS profiling) is an emerging approach based on rapid and high-throughput screening of ions from molecules directly detected in biological samples, including intact cells and crude extracts (Albrethsen, 2011). Although MALDI-TOF MS profiling is one of the most promising mass spectrometry techniques, it detects only the most intense molecular ions of low molecular mass (typically < m/z 20,000) and does not enable direct protein identification. MALDI-TOF MS profiling commonly leads to the detection of a large number of biomarkers and captures the fingerprints of cells, tissues, and biological fluids under normal or altered conditions. MALDI-TOF MS profiling has been initially targeted for the classification of clinically relevant medical applications, including cancer (Sidransky et al., 2003) and pathogenic microorganism (Ilina et al., 2009) diagnostics. Recently, it has proved to be a versatile tool toward an unprecedented number of applications. Clinical bacterial (Eigner et al., 2009) and mycobacterial (Oswald-Richter et al., 2012) isolates, entomopathogenic soil fungi (Lopes et al., 2014), environmental yeasts (Agustini et al., 2014), and viruses (Calderaro et al., 2014) are some of the biological systems which were identified and clustered by MALDI-TOF MS profiling. In addition, it has been applied for quality control of foods including honey (Wang et al., 2009) and chocolate (Bonatto and Silva, 2014a).

Interestingly, it has also been demonstrated by several authors that MALDI-TOF MS profiling can exhibit resolution even toward bacterial strain-level identification (reviewed in Sandrin and Goldstein, 2013). MALDI-TOF MS profiling has also been applied for the detection of methicillin-resistant strains of *Staphylococcus aureus* (Sparbier et al., 2013). Recently, Bonatto and Silva (2014b) reported the use of this technique for the differentiation of yeast cultures submitted to metal nanoparticle stress, showing that this method can have additional applications beyond microorganism species identification. We have further tested MALDI-TOF MS profiling for bacterial and plant samples and also successfully differentiated specific biological conditions (unpublished data).

The main prerequisite for improved identification of biological samples by MALDI-TOF MS profiling is a curated database of mass spectra. Commercial and public mass spectral libraries typically contain hundreds to thousands of entries which are clustered according to a subject category or biological taxa. Although there are several companies manufacturing MALDI instruments, there are only three major commercial systems available in the market (BioTyper—Bruker Daltonics, SARAMIS—Shimadzu and Anagnostec, and MicrobeLynx™—Waters Corporation) for which, equipment, software, and database are integrated aiming at the identification and classification of organisms. These systems typically apply robust algorithms that are associated with multivariate statistic approaches and show distinct quantitative levels of reliability. Indeed, some authors have compared the results obtained using two of the above mentioned approaches and their performances were overall similar (Lohmann et al., 2013).

## MALDI-TOF MS profiling in plant proteomics

The use of MALDI-TOF MS profiling in plants has been restricted to metabolite profiles and has been rarely reported (Fraser et al., 2007). Therefore, to date there is no database sharing spectra from protein profiles of plant tissues or organs under physiological or altered states. However, the application of this technique can have high impacts in plant proteomic studies, contributing for sample differentiation and/or protein marker discovery in agriculture and industry. The identification of biological markers is an important field of study and can significantly help breeders select cultivars better adapted to diverse biotic and abiotic stresses or in different developmental stages.

One of the main challenges in applying MALDI-TOF MS profiling to plant studies is the high complexity observed in plant tissues. For example, leaves have a high ribulose-1,5-bisphosphate carboxylase/oxygenase (RuBisCO) content, which seems to interfere in the reproducibility of protein profiles. We have analyzed *Brassica oleracea* leaves infected with the bacterium *Xanthomonas campestris* pv. *campestris* in comparison to non-infected leaves and found that the results did not always show a clear distinction and sometimes clustered different samples together (unpublished data). Reproducibility limitations, specially related to peak intensities, have also been reported in human disease diagnosis when using MALDI TOF MS (Albrethsen, 2007). However, we are confident that a fine tuning of the methodology may be able to solve this problem. Sample preparation and tissue origin, for example, may be crucial for the high reproducibility required during MALDI-TOF MS profiling experiments. The removal of highly abundant proteins such as RuBisCO may result in more reliable and consistent results. In addition, plant organs and tissues showing more constant proteomes (e.g., pollen and seeds) could be preferable when the molecular signature of a given species is the most important challenge. On the other hand, leaves and other more variable plant organs must be the choice if the main interest is to evaluate a specific biological state.

Another challenge is the identification of the proteins detected in the MALDI-TOF MS profiles since this approach is merely comparative. One possibility is the use of enzymatic hydrolysis to generate peptide fingerprints representing a given physiological stage. We recently used this approach in order to generate a representative pool of the peptides hydrolysable by trypsin from the *B. oleracea* proteins and further detected as a shotgun by the MALDI-TOF MS profiling approach. However, the proteins containing the peptides detected in the MALDI-TOF MS profiling could not be inferred based only on predicted peptide masses obtained by the hydrolysis due to the low mass accuracy and mainly the signal suppression associated with the high abundance of some molecular components.

The species identification based on MALDI-TOF MS profiling approaches is based on ribosomal proteins, which are enriched by the extraction method with formic acid. Indeed, for accurate identification purposes, this enrichment is crucial. However, when other extraction methods are used, it is possible to get a broader range of protein types, allowing a more global view of the proteins being expressed. We have already tested phenol extraction method followed by precipitation in ammonium acetate and methanol for bacterial and plant samples and have found that a higher diversity of proteins was obtained. Therefore, when using this approach for protein expression profiling, the use of such extraction methods seems preferable. It is noteworthy that ions from other biological molecules (e.g., lipids and secondary metabolites) and mass ranges (e.g., lower than m/z 1000) can be used for identification of biological conditions. Ion sources that do not require conventional MALDI matrices or approaches that are based on surface-enhanced laser desorption/ionization (SELDI) or matrix-free methods are being currently developed by researchers and companies worldwide and forthcoming years will show significant evolution.

Overall, MALDI-TOF MS profiling is a powerful technique that can have various potential applications in plant proteomics, such as protein marker discovery, and provide substantial contributions for genetic breeding programs and biotechnology. Efforts need to be made in order to adapt the methodology for different plant tissues, however, our studies have shown that the use of this technology is feasible, fast and reliable and can be successfully applied in plant proteomic studies.

### Conflict of interest statement

The authors declare that the research was conducted in the absence of any commercial or financial relationships that could be construed as a potential conflict of interest.
